# Schwannoma-like thoracic glomangioma: case report

**DOI:** 10.1093/jscr/rjab527

**Published:** 2022-08-05

**Authors:** Eyüp Çetin, Ümmügülsüm Sariçiçek

**Affiliations:** Department of Neurosurgery, Faculty of Medicine, Van Yüzüncü Yıl University, 65000, Van, Turkey; Department of Neurosurgery, Faculty of Medicine, Van Yüzüncü Yıl University, 65000, Van, Turkey; Department of Pathology, Faculty of Medicine, Van Yüzüncü Yıl University, 65000, Van, Turkey

## Abstract

In this study, we present an extremely rare case of thoracic spinal glomangioma located at the thoracic T7–8 level in the foraminal region, mimicking a schwannoma tumor, in a 65-year-old female patient who was admitted to our clinic with a 6-month history of back pain and weakness in legs.

## INTRODUCTION

Benign tumors that grow gradually over a long period of time cause the body to allow for intruders. Various soft tissue, bony and neural modifications occur as a result of compressive effects of the tumor [1]. Foraminal sheath tumors are generally either neurofibroma or schwannoma and comprise 40% of all extramedullary tumors [2]. Intradural tumors account for 75% and 24.6% of spinal cord tumors in adults and children, respectively [3]. Among spinal tumors, the most common extra-axial tumors include schwannoma (47%), meningioma (16%), neurofibroma (2%), and malignant peripheral nerve tumor (<1%) [4]. Glomangioma, also known as glomus tumor, is a benign tumor mostly found in the extremities and very rarely in the spine [5]. A normal glomus body is an arteriovenous component of the dermis layer of the skin, involved in thermoregulation [6]. Literature indicates that most primary spinal glomus tumors show osteolytic patterns [7]. In this study, we present a case of thoracic spinal glomangioma localized to the foraminal region at the thoracic T7–8 level, mimicking a schwannoma.

## CASE PRESENTATION

**Figure 1:**
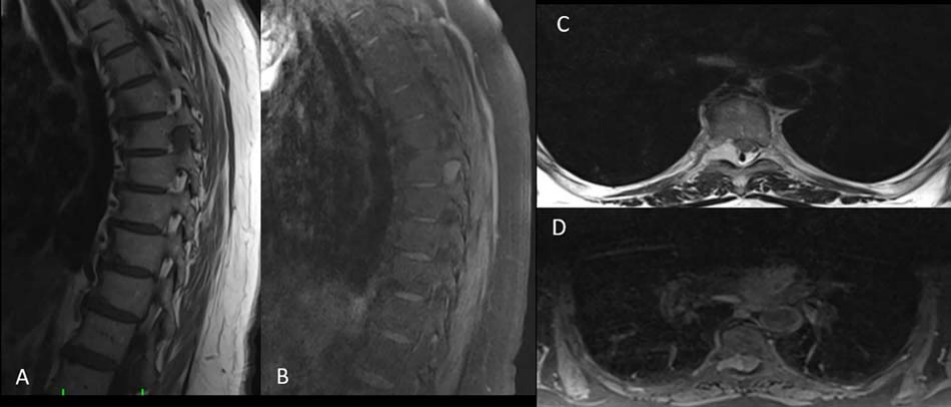
On preoperative MRI, the mass was (**A**) hypointense on T1-weighted sagittal images, (**B**) hyperintense on contrast-enhanced T2-weighted sagittal images, (**C**) hyperintense on T2-weighted axial images and (**D**) isointense on T1-weighted axial images.

A 65-year-old female patient was admitted to our clinic with the complaints of back pain and progressive weakness in her legs for the last 6 months. An ethics approval and consent to participate were not applicable since the present study is a report about the experiences and observations of medical practices associated with a single individual. On physical examination, the patient had paresthesia and hyperreflexia in both legs.There was no bladder dysfunction or loss of motor strength, while there was bilateral loss of vibratory sense below T8 and moderate loss of pinprick. Bilateral knee clonus and the Babinski sign were negative. Thoracic magnetic resonance imaging (MRI) showed a mass lesion mimicking a meningioma or schwannoma at the foraminal level of the right T7–T8 vertebrae. The tumor was hypointense on T1-weighted images and hyperintense on T2-weighted images in both contrast and noncontrast-enhanced images (Fig. 1). The patient was operated on due to progressive paresthesia and weakness and T7 hemilaminectomy was performed. The dura mater and tumor margins were evident. The transition between tumor tissue and spinal cord tissue was slightly difficult to establish superficially. The tumor tissue was vascular in nature; therefore, the mass was shrunk with bipolar forceps and was completely excised before it was allowed to bleed. No tumor extension was found ventral to the T7 level. Dural leakage developed during tumor excision. Duraplasty was performed by suturing the injured dura and the dura was supplemented with fibrin glue (Tisseel®; Baxter, Newbury, Berkshire, UK).

**Figure 2:**
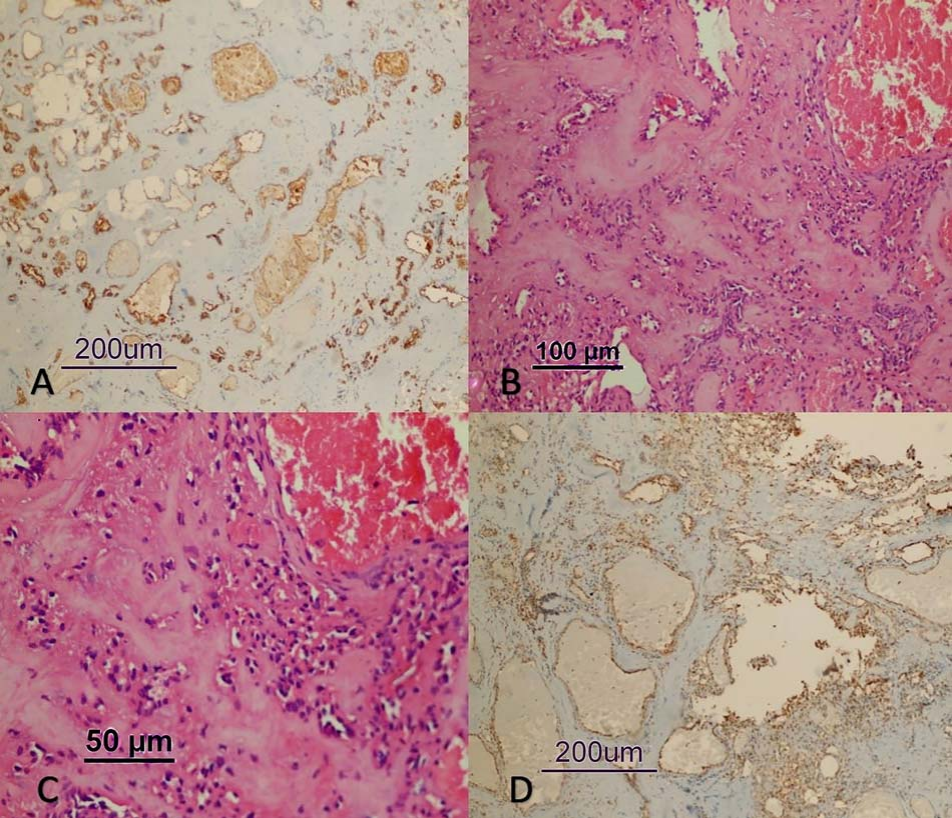
Histological sections of the tumor detected in the patient (CD31 [×100] [**A**]), (H&E [×200] [**B**]), (H&E [×400] [**C**]), (SMA [×100] [**D**]).

Immunohistochemical examination indicated the following features in neoplastic cells: CD34 (+), CD31 (+), SMA (+), P53 (−), EMA (−), PanCK (−), Progesterone (−), S100 (−), PAS (−), CEA (−), GFAP (−), Synaptophysin (−) and Chromagranin (−). Additionally, Ki-67 proliferation index was determined as <1% (Fig. 2). Based on these features, the mass was diagnosed as degenerative glomangioma.

## DISCUSSION

Glomus tumor is mostly seen in hands, particularly in the nail beds [8]. The rate of successful total excision in glomus tumor of hands is remarkably high (95%) [9]. Moreover, its recurrence rate over the 2-year follow-up is remarkably low (~5%). Extradigital glomus tumor is highly rare in the literature and primary or metastatic extradigital glomangiomas are also rare [10].

In a review by Zheng *et al.*, only 13 cases were reported [11]. In MRI Examinations, these lesions are ovoid and well defined, and are illustrated hyperintensity or isointense on T1- and hyperintense on T2-weighted MRI images [12]. Most authors have reported that surgery is indicated in cases of spinal cord compression. Embolization, ligation or radiotherapy and surgery are treatments used for the glomus tumors. With the total resection of tumor has reported a satisfactory outcome without recurrence at the 5-year follow-up [14]. In the case presented, we showed a degenerated thoracic glomus tumor originating from the thoracic foramen, compressing the medulla spinalis and radiologically mimicking a spinal meningioma or spinal schwannoma. We consider that spinal glomus tumor is rarely encountered by spinal surgeons and thus should be kept in mind.

## CONCLUSION

In patients presenting with a mass compressing the medulla spinalis, glomus tumor should be suspected even if the lesion mimics a spinal meningioma or schwannoma.
